# Application of a Medical Diode Laser (810 nm) for Disinfecting Small Microbiologically Contaminated Spots on Degraded Collagenous Materials for Improved Biosafety in Objects of Exceptional Historical Value From the Auschwitz-Birkenau State Museum and Protection of Human Health

**DOI:** 10.3389/fmicb.2020.596852

**Published:** 2020-12-18

**Authors:** Dorota Rybitwa, Anna Wawrzyk, Mansur Rahnama

**Affiliations:** ^1^Preservation Department, Auschwitz-Birkenau State Museum, Oświęcim, Poland; ^2^Sanitary-Epidemiological Station, Kraków, Poland; ^3^The Chair and Department of Oral Surgery, Medical University of Lublin, Lublin, Poland

**Keywords:** diode laser, microorganisms, disinfection, biodeterioration, human health, collagenous materials, historical leather shoes, Auschwitz-Birkenau State Museum

## Abstract

The research aim was to optimize the operating parameters of a diode laser irradiation for the effective disinfection of degraded collagenous materials. Historical leather shoes stored at the Auschwitz-Birkenau State Museum in Oświęcim (Poland) were the main study objects. Surfaces of contaminated small spots occurring on the degraded materials were sampled with moistened swabs and microbiologically examined using the molecular techniques MALDI-TOF MS, 16S rRNA, and NGS sequencing. The surfaces were colonized by bacteria with 10^6^ CFU/100 cm^2^ and 10^4^ CFU/100 cm^2^ by fungi, on average. Microorganisms of the genera *Bacillus* and *Penicillium* were predominant. The effectiveness of the laser treatment was assessed for the new and degraded collagenous materials against isolated environmental strains using four variants of exposure time and number of repetitions. 0.3 W/CW 2 × 2 min variant was the most effective and also did not noticeably change the color of the treated samples. The variant caused a reduction in the numbers of microorganisms by 96–100%. After 1 month, four types of leather were subjected to comprehensive physico-chemical analyses. SEM and FTIR techniques confirmed that laser irradiation in the selected optimal variant did not affect the surface morphology and collagen structure, while XPS technique enabled detection of subtle changes in non-historical protective coatings on the surfaces of tested degraded historical materials.

## Introduction

KL Auschwitz-Birkenau, a former German Nazi Concentration and Extermination Camp in Oświęcim, is evidence of one of the greatest crimes ever committed against humanity. Initially it served for the annihilation of prisoners of various ethnicities through inhuman conditions such as starvation and slave labor. Later it became the largest extermination center established by the German Nazi regime for the immediate and mass killing of people. The historical collection, which is currently located at the Auschwitz-Birkenau State Museum in Oświęcim, Poland (A-BSM), retains the memory of people who were deliberately murdered in this place and illustrates the way in which it was done. Personal items of the victims stored in the collection constitute a testimony of their lives before deportation to the extermination camp and show the way in which their possessions were used. Due to the careful preservation of the original evidence carried out without unnecessary reconstruction, the place and the objects are still authentic and integral.

More than 100,000 pieces of the several hundred thousands of objects stored at the A-BSM are footwear. These are casual, work, and formal shoes for women, men, and children. Boots and ankle boots dominate, but pumps and woven sandals are found in large quantities as well. Most of them are made of grain leather, with a few made of suede leather, and some contain textile elements. The upper parts of the shoes are made mostly of chrome-tanned leather, and some are vegetable-tanned. Inside the objects, cardboard insoles trimmed with leather and fabrics are often found. All the shoes are distorted, worn down, cracked, and contaminated. The impurities rubbed into the linings and particles of mud, soil, or sand embedded in the outsoles of the shoes partly come from the period of their use by prisoners in the camp. From the early 1960s the objects underwent conservation treatment, and therefore they survived to this day. Notes from the conducted works show that they were cleaned in baskets with the use of water, sawdust, and sand, and then disinfected several times with an alcohol solution of p-chloro-m-cresol (PCMC), greased with a mixture of lanolin, petroleum ether, beeswax, and cedar oil, and finally sprayed with varnish: a toluene solution of poly (methyl methacrylate) or an aqueous solution of poly (vinyl alcohol). In 2005 new procedures were introduced, i.e., the cleaning of dust with brushes and vacuum cleaners, the removal of more strongly bounded dirt using tampons soaked in water with a solution of Marseille soap, and greasing with a mixture of neatsfoot oil, lanolin, white spirit, and ethanol.

When leather products are stored under adverse conditions, they may undergo biodeterioration because of the content of natural introduced both during the production process and during conservation treatment. At first, tannins, fats, and simple proteins contained in the leather are broken down, followed by the breakdown of pure collagen (Strzelczyk, 2004; Strzelczyk and Karbowska-Berent, 2004). These components constitute substrates for the development of microorganisms. Most often, the germination of fungal spores and bacterial growth begins in a few small places. Then bacteria and fungi may spread to adjacent surfaces on the object or infect other objects. Since the museum was established, microbial contamination of a significant number of shoes was observed and confirmed thrice by laboratory analyses. Therefore, during each inspection of the collection, special attention is paid to the presence of microbial contamination in the form of small spots, which allows actions to inhibit microbial development to be implemented at the right time.

**FIGURE 1:**
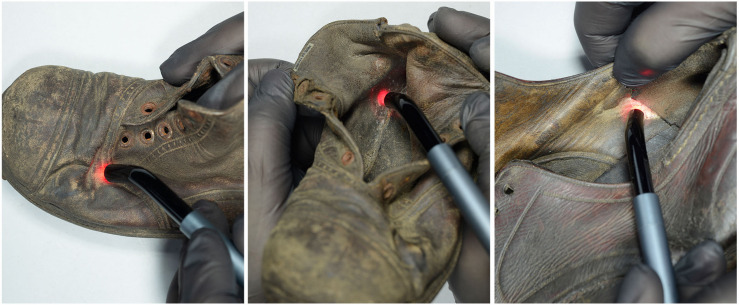
Laser irradiation of small spots on historical leather shoes (author: Margrit Bormann).

The disinfection of historical objects made of collagenous materials, including leather shoes, is a very important stage in conservation treatments. It eliminates vegetative forms of microorganisms that contribute to biodeterioration of materials and at the same time pose a threat to the objects and to human health. Disinfectants applied in museums must be sufficiently effective. Additionally, they cannot cause changes to the properties of treated materials that have already been heavily degraded by previously used substances and external factors. Methods for decontaminating museum objects, which were tested and developed at the A-BSM, i.e., vaporized hydrogen peroxide (VHP) or ethylene oxide (EtO), require treatment of the entire object. Other methods also used or tested in the field of conservation include gamma irradiation, X-ray irradiation, and misting with silver nanoparticles (AgNPs) (Cortella et al., 2011; Gutarowska et al., 2014; Vadrucci et al., 2019). Such comprehensive disinfection is not always necessary, because microbial contamination often occurs locally on small surfaces (spots). To date, spraying with 70% ethanol is the only method for disinfecting small spots on historical leather. However, it was rarely used at the museum because aqueous solution is needed for this purpose and as a result of leather wetting it may shrink, stiffen, or become brittle or stained (Dignard and Mason, 2018). Therefore, efforts are being made to find other techniques which will allow for local elimination of microorganisms, and at the same time will be safe for objects containing degraded collagenous materials, for employees, and for the environment. Laser irradiation using specially adapted settings may prove to be such a disinfection method (Figure 1).

The aim of the study was to optimize the operating parameters of a medical diode laser with a wavelength of 810 nm for effective decontamination of very small, microbiologically contaminated spots occurring on degraded collagenous materials. Additionally, analogical tests were performed on new collagenous materials for comparison. In the beginning, the quantitative and qualitative assessment of microbiologically contaminated surfaces of chrome-tanned and vegetable-tanned grain leathers from historical shoes stored at the A-BSM was performed to detect microbial communities inhabiting these materials. Generally, standard culture-dependent methods involving culturing, characterization, and phenotypic classification of microorganisms using selective microbiological media, which favor a desired microbial group, are insufficient. Therefore, non-selective media for cultivation and molecular methods based on MALDI-TOF MS and 16S rRNA gene nucleotide sequences comparison were used in this study to identify culturable microorganisms. Moreover, culture-independent molecular methods with next-generation sequencing (NGS) were utilized to better understand taxonomy and diversity of microorganisms inhabiting the degraded collagen-based materials, including bacteria and fungi in a viable but non-culturable (VBNC) state. Next, biocidal effectiveness of laser irradiation and its impact on the optical properties of various types of leather were tested to choose the best configuration of parameters for disinfection. The following parameters of laser irradiation were examined: power of 0.3 W, continuous wave mode, two variants of exposure time (30 s and 2 min), and two variants of number of repetitions (single and double application). Microbiological tests were carried out on the new (model) and degraded (historical) leathers inoculated with four and 11 microbial species, which were the most abundant, respectively. Spectrophotometrical color measurement of two model and two historical leathers differing in tanning method were taken to assess visual alterations after laser treatment. Similarly, as with most collagen-based materials, leather may undergo degradation resulting from disinfection, that may proceed according to the mechanisms of oxidation, hydrolysis, or denaturation. Therefore, the effect of laser irradiation on the physico-chemical properties of the leathers was assessed 1 month after treatment with the use of a variant which was the most biocidally effective and did not noticeably change the color of the materials. In order to minimize the necessity of destruction of the objects and the size of taken samples, instrumental analyses were chosen. Scanning electron microscopy (SEM), infrared spectroscopy (FTIR), and X-ray photoelectron spectroscopy (XPS) were utilized to monitor morphological, structural, and chemical alterations. These comprehensive analyses allowed the evaluation of possible variations in the secondary and tertiary structures of collagen and conditions of layers covering historical objects and thus degradation of tested leathers due to laser irradiation.

## Materials and Methods

### Collagenous Materials

Historical shoes (number of samples, *N* = 30) from the A-BSM collection, dated to the first half of the 20th century, were subjected to the quantitative and qualitative assessment of microbiological contamination. The shoes were previously stored in display cases at the exhibition, in which environmental conditions cannot be regulated. Swabs were taken from small spots on the external surfaces of shoes made of the following degraded collagenous materials, grain chrome-tanned leather (ctl, *N* = 15) and grain vegetable-tanned leather (vtl, *N* = 15), which had visible signs of biodeterioration.

Two types of grain leather from the historical shoes, which were provided by conservators from the A-BSM, and corresponding types of contemporary model leathers were used to assess biocidal effectiveness and effect of laser irradiation on properties of the collagen-based materials. Historical brown chrome-tanned leather from the shoe quarter and historical brown vegetable-tanned leather from the shoe counter represented degraded collagenous materials. Model black chrome-tanned leather and model natural vegetable-tanned leather represented new collagenous materials. All four types of leather were subjected to color measurements, SEM, FTIR, and XPS analyses. Due to the large amount of material which is required for tests of antimicrobial efficacy and the very limited availability of historical leathers, only one type of historical collagen-based material and the corresponding type of model material were selected for these tests (vegetable-tanned leather).

### Quantitative and Qualitative Assessment of Microbiological Contamination

Microbiological contamination of tested leather shoes from the A-BSM was assessed quantitatively using plating methods and qualitatively with various methods. Moistened cotton swabs (0.85% NaCl), which were used to take samples from 1 cm^2^ of tested surfaces, were immersed in 10 ml of sterile peptone water (0.10% peptone and 0.85% NaCl) and shaken for 1 h (initial suspensions). Then, serial decimal dilutions and plating using pour plates (for quantitative assessment) and spread plates (for qualitative assessment with molecular culture-dependent methods) techniques were performed with the use of the following microbiological media: TSA (Tryptic Soy Agar, Merck, Germany) with nystatin for bacteria and Sabouraud Agar (Merck, Germany) with chloramphenicol for fungi. The plates were incubated for 2 days at a temperature of 30 ± 2°C (bacteria) and 5 days at a temperature of 25 ± 2°C (fungi). The microbial colonies grown on the plates inoculated with the pour plate method were counted and the results were presented as Colony Forming Units per 100 cm^2^ of tested surface (CFU/100 cm^2^). The microbial colonies grown on the plates inoculated with the spread plate method were subjected to species identification.

The following culture-dependent molecular methods were used to identify bacteria: matrix-assisted laser desorption/ionization time-of-flight mass spectrometry (MALDI-TOF MS) and comparison of the nucleotide sequences of 16S rRNA genes. Samples of all strains were prepared with extended direct transfer method and underwent identification by MALDI-TOF MS. From the strains that could not be identified to the species level or from which an uncertain result was obtained using the first method, 16S rRNA genes were isolated using mechanical cell lysis and minicolumn methods and compared with sequences published in the National Center for Biotechnology Information (NCBI) database. In the case of fungi macroscopic and microscopic observations, collation with data in diagnostic keys were employed. Moreover, culture-independent molecular methods based on next-generation sequencing (NGS) of the 16S rRNA gene and ITS1 region on Illumina platform was used to carry out metagenomic analyses aiming at the detection of the complete microbial community inhabiting the historical shoes. Total genomic DNA necessary for sequencing was manually extracted using the minicolumn method combined with the isopropanol precipitation from mixtures of all initial suspensions of the microorganisms sampled from particular types of the historical leathers. The abovementioned qualitative assessment was performed using devices and methodology presented by Wawrzyk et al. (2020). Moreover, the following criteria for the validity of the results of identification were taken into account: score value ≥ 2.0 (for MALDI-TOF MS), sequence identity ≥ 98% (for 16S rRNA gene comparison), and relative abundance ≥ 2% (for NGS).

### Laser Irradiation

In this study a medical diode laser emitting radiation at a wavelength of 808 ± 10 nm characterized by high power accuracy and stability with a handpiece containing 8 mm diameter light guide making a collimated laser beam was used. During the irradiation (treatment) the handpiece was positioned perpendicularly at a distance of approximately 1–2 mm from the samples, which were placed on sterile microscope slides. The samples were irradiated with laser using the following parameters: 0.3 W power and continuous wave (CW) mode, named together as 0.3W/CW. Within the scope of the assessment of biocidal effectiveness and impact on the optical properties, various configurations of exposure time and number of repetitions of laser application were examined: single and double irradiation for 30 s (named as 1 × 30 s and 2 × 30 s, respectively) and single and double irradiation for 2 min (1 × 2 min and 2 × 2 min, respectively). In the case of double treatment, a 2 h break was applied. Based on this, the most optimal variant was selected, i.e., 0.3 W/CW 2 × 2 min, and then it was tested for possible changes in the physico-chemical properties of the leathers. Laser fluence and power density of the 0.3 W/CW 2 × 2 min variant were 72.0 J/cm^2^ and 0.6 W/cm^2^, respectively.

### Tests of Biocidal Effectiveness

The assessment of the antimicrobial efficacy of laser irradiation was carried out against these environmental strains, which were isolated from all tested leather shoes with the highest frequency and belong to species that pose a threat to the condition of objects and/or human health. Samples of the model and historical leathers were inoculated with four and 11 environmental strains, respectively. Fewer strains of microorganisms (only those commonly found on surfaces of various objects and rooms at the A-BSM, unpublished data) were tested in the case of the model leather than historical one, because it is not a main subject of this study. Moreover, studies using historical leather were extended with the following strains from the American Type Culture Collection: *B. subtilis* WDCM 00003 and *A. niger* WDCM 00053. These strains were chosen because the first is known for its high resistance to thermal disinfection (Setlow, 2006) and the second spreads at the A-BSM the most easily (unpublished data).

The tests of the biocidal effectiveness of laser irradiation were performed in triplicate. Samples of materials constituted 8 mm discs aseptically cut from the model and historical leathers. Inocula containing 10^4^ bacterial cells or fungal cells and spores in 1 ml of sterile saline (0.85% NaCl) were prepared. Their concentration was established using densitometric and plate methods. Leather discs were inoculated with 50 μl of inocula of individual microorganisms to obtain a microbial concentration in the samples similar to that of the historical leather shoes. After 2 h drying inside a laminar flow chamber, test samples were irradiated with the laser using proper operating parameters. Control samples were kept unirradiated. To determine the biocidal effect of laser treatment over many cell cycles of microbial growth (periods determined empirically as the ones giving the best percentage reduction in the microbial numbers, unpublished data) bacterial and fungal numbers were assessed after 1 and 2 days of incubation of treated and untreated samples at a temperature of 22 ± 2°C. The numbers of microorganisms in laser-treated (*N*_*t*_) and untreated (*N*_*unt*_) samples were determined with the same methodology as for the assessment of the microbiological contamination of the historical shoes. Then they were used to calculate the percentage reduction in microbial number (*R*) resulting from laser irradiation according to the equation:

$$R=\left(\frac{N_{u\,n\,t}-N_{t}}{N_{u\,n\,t}}\right)\cdot 100\%$$

### Assessment of Changes in the Optical and Physico-Chemical Properties

The effect of laser irradiation on the color, morphology, structure, elemental composition, and chemical condition of the leathers was assessed after 1 month of laser treatment using the devices and methodology presented by Wawrzyk et al. (2020), supplemented with additional aspects described below.

Spectrophotometrical color measurements were taken in exactly the same place of the sample before and after laser treatment. The values of variations in individual color coordinates (*ΔL^∗^, Δa^∗^, Δb^∗^*) and total color change (Δ*E*^∗^) resulting from irradiation were calculated in accordance with colorimetric guidelines of the Commission on Illumination (CIE, 2018). To assess the color change, criteria described by Mokrzycki and Tatol (2011) were used, taking into account precision of the color measurement achieved in the laboratory. It was concluded that Δ*E^∗^* < 0.87 CIE*L^∗^a^∗^b^∗^* units have no difference between two colors, and Δ*E^∗^* < 1.74 CIE*L^∗^a^∗^b^∗^* units have no change in color noticeable by an unexperienced observer, which constitutes an acceptable result in the field of conservation of cultural heritage.

For subsequent instrumental analyses, two sets (unirradiated and irradiated with laser) of samples of 0.5 cm^2^ were necessary. One of them was used for Fourier transform infrared spectroscopy (FTIR) and scanning electron microscopy (SEM), and the second only for X-ray photoelectron spectroscopy (XPS). Morphological changes on the surfaces of the leather and collagen fibers were assessed based on microscopic images taken at magnifications of 100 × and 5,000 × (SEM). Modifications in the structure, elemental composition, and chemical condition of the samples were analyzed using FTIR and XPS spectroscopies. The use of these two techniques was motivated by the fact that during laser irradiation the outermost layer of the material is most exposed to heating and consequently to possible damage (that can be detected by XPS measurements, which are taken from a superficial layer with a thickness of 2–5 nm). However, changes in temperature and physico-chemical properties may also occur in deeper layers (for which FTIR measurements taken from a layer of 2–3 μm were used). FTIR analyses consisted of a preliminary comparison of appropriate spectra and determination of variations in relative intensities (IA_I_/A_II_) and wavenumber shifts [Δν (A_I_ -A_II_)] of two diagnostic bands of collagen infrared spectrum related to the process of leather degradation: amide I band (A_I_, νC = O stretching vibration related to the protein backbone) falling in a region between 1,648 and 1,632 cm^–1^, and amide II band (A_II_, νC–N, δN–H vibration, depending on the conformation adopted by the peptide) located near 1,545 cm^–1^. Criteria developed by Vyskočilová et al. (2019) were utilized to assess the damage in leather occurring in accordance with mechanisms of oxidation and hydrolysis, which are visible in the FTIR-ATR spectra. The XPS analyses included determination of variations in elemental composition, degree of oxidation, and surface polarity. The values of the ratio of C:O atoms were calculated based on C1s and O1s spectral bands (survey spectra). The degree of oxidation was estimated as the C_oxid_:C_unoxid_ ratio, i.e., ratio of the sum of oxidized forms of carbon atoms (bands: C1sC–C1sG) to the sum of unoxidized (aliphatic and aromatic) forms of carbon (C1sA and C1sB). Surface polarity was estimated as the O_carb_:O_other_ ratio, which was calculated based on the sum of carbonyl groups (O1sA and O1sB) and the sum of other oxygen-containing forms of carbon (O1sC–O1sE).

### Assurance of Research Quality

To assure quality in the studies, precision in the repeatability conditions was defined for all quantitative methods: 0.2 log for microbial number, 13% for color measurement spectrophotometry, 1% for FTIR, and 7% for XPS. Moreover, all samples were measured several times: twice for determination of microbiological contamination and biocidal effectiveness, thrice for analysis of optical properties, and four times for imagining of morphology. Samples for assessment of structure, elemental composition and chemical condition were scanned as many times as required to obtain high quality of collected spectra.

### Statistical Analysis

The arithmetic mean and standard deviation were calculated for the numbers of microorganisms constituting results of tests of microbiological contamination and biocidal effectiveness of laser irradiation. One-way analysis of variance (ANOVA) and least significant difference (LSD) tests at a significance level of *p* < 0.05 were used to assess statistical significance of differences in microbial numbers between unirradiated and irradiated samples (^∗^) and between samples treated with various variants of laser operating parameters (^#^ and ^$^). The analyses were performed using STATISTICA 6.0 software (Statsoft, United States).

## Results

### Microbiological Contamination of the Historical Shoes From the A-BSM

Microbiological contamination of tested spots on the historical leather shoes made of chrome-tanned leathers (ctl) was comparable to that of vegetable-tanned leathers (vtl, Table 1). Bacteria contaminated the shoes to a greater extent than fungi. A slightly higher than average number of bacteria was detected on the surfaces of chrome-tanned leathers than on the vegetable-tanned ones. An inverse relationship was noted for numbers of fungi inhabiting both types of leather. Most of the spots on the historical objects (ctl: 73.33% and vtl: 80.00%) showed bacterial contamination at the level of 10^6^ CFU/100 cm^2^. Fungi were detected in approximately half of the samples (ctl: 53.33% and vtl: 60.00%). In these samples, concentration at the level of 10^4^ CFU/100 cm^2^ was found.

**TABLE 1:** Quantitative microbiological contamination of the spots on the historical leather shoes at the A-BSM.

Microorganisms	Average concentration of microorganisms on the materials [CFU/100 cm^2^]	
	Chrome-tanned leathers	Vegetable-tanned leathers
Bacteria	7.61 × 10^6^ ± 9.95 × 10^6^	1.96 × 10^6^ ± 1.43 × 10^6^
Fungi	2.33 × 10^4^ ± 2.26 × 10^4^	2.56 × 10^4^ ± 2.50 × 10^4^

**mean ± standard deviation.**

The use of culture-dependent molecular methods allowed the identification of 27 species of bacteria and 12 species of fungi isolated from the A-BSM leather objects (Table 2). Among the identified bacteria, the most numerous were gram-positive bacilli belonging to the genus *Bacillus* (17 species) and related bacteria of the genera: *Oceanobacillus* (1), *Ornithinibacillus* (1), *Paenibacillus* (1), and *Psychrobacillus* (1). Additionally, gram-positive cocci of the genera *Staphylococcus* (3) and *Micrococcus* (1), and gram-negative bacteria *Acinetobacter* (1) and *Pantoea* (1) were detected as well. Most of the identified fungal species belonged to the family Trichocomaceae (*Aspergillus, Paecilomyces*, and *Penicillium*: eight species), while families *Aureobasidium, Cladosporium, Geotrichum*, and *Hormographiella* were represented by a single species. The following bacteria–*A. lwoffii, B. cereus, B. licheniformis*, *B. pumilus, B. subtilis, M. luteus, P. agglomerans*, and *S. epideremidis*–and fungi–*A. flavus, A. niger, C. cladosporioides, P. variotii, P. chrysogenum, and P. vermiculatum*–were detected on both types of leather. *B. cereus* was the only microorganism found in all tested samples.

**TABLE 2:** Results of the identification of microorganisms detected on the spots on the historical leather shoes using culture-dependent molecular methods.

Microbial species	Occurrence of microorganisms		NCBI Id / Accession no.	SV / S
	Chrome-tanned leathers	Vegetable-tanned leathers		
**Bacteria**				
*Acinetobacter lwoffii*^1^	+	+	28090	1.85–2.12
*Bacillus amyloliquefaciens*^1^	+		279145	1.73–1.99
*Bacillus aquimaris*^2^	+		MF506765.1	99.87
*Bacillus cereus*^1^	+	+	1,396	1.83–2.08
*Bacillus endophyticus*^1^	+		135735	1.80
*Bacillus glycinifermentans*^2^	+		MK403913.1	99.61
*Bacillus haynesii*^2^		+	MK680081.1	99.72
*Bacillus licheniformis*^1^	+	+	1,402	1.75–2.18
*Bacillus megaterium*^1^	+		1,404	1.90–2.03
*Bacillus mojavensis*^1^	+		72,360	1.89
*Bacillus oceanisediminis*^1^	+		1,386	2.07–2.16
*Bacillus oleronius*^1^	+		38,875	1.92
*Bacillus pumilus*^1^	+	+	1,408	1.73–1.87
*Bacillus simplex*^1^	+		1,478	1.70
*Bacillus sonorensis*^2^		+	KX447610.1	99.81
*Bacillus subterraneus*^2^		+	MK571199.1	100.00
*Bacillus subtilis*^1^	+	+	135461	1.95–2.16
*Bacillus thuringiensis*^1^	+		1,428	1.95
*Micrococcus luteus*^1^	+	+	1,270	2.12–2.34
*Oceanobacillus picturae*^2^	+		MK063866.1	99.65
*Ornithinibacillus californiensis*^2^		+	MH016376.1	99.88
*Paenibacillus lautus*^2^	+		CP032412.1	99.67
*Pantoea agglomerans*^1^	+	+	549	2.10–2.36
*Psychrobacillus psychrodurans*^1^		+	126157	1.76
*Staphylococcus epidermidis*^1^	+	+	1,282	2.07–2.09
*Staphylococcus haemolyticus*^1^	+		1,283	1.83
*Staphylococcus warneri*^1^	+		1,292	2.02
**Fungi**				
*Aspergillus flavus*	+	+		
*Aspergillus niger*	+	+		
*Aureobasidium pullulans*		+		
*Cladosporium cladosporioides*	+	+		
*Geotrichum candidum*	+			
*Hormographiella verticillata*	+			
*Paecilomyces formosus*		+		
*Paecilomyces variotii*	+	+		
*Penicillium chrysogenum*	+	+		
*Penicillium citrinum*		+		
*Penicillium variabile*		+		
*Penicillium vermiculatum*	+	+		

**+–microbial species detected in the samples; ^1^–bacterial species identified using MALDI-TOF-MS; ^2^–bacterial species identified using 16S rRNA gene sequence comparison; NCBI Id–sequence record processed by NCBI (for ^1^); Accession no.–GenBank, NCBI 16S rRNA gene sequence comparison (for ^2^); SV–the level of similarity between an unknown tested specimen and a reference species indicated by a log(score) (for ^1^); S–similarity to compared 16S rRNA gene sequence [%] (for ^2^)**

As a result of NGS sequencing, raw read pairs of V3–V4 16S rRNA gene sequences (ctl: 100636 and vtl: 84563) and ITS1 region sequences (ctl: 53052 and vtl: 38147) were obtained. Bioinformatic analyses of 16S rRNA sequences enabled the classification of 50.73% (ctl) and 26.46% (vtl) reads in total. For tested spots on both types of leather, more than 99.60% of classified reads were assigned to the kingdom Bacteria (50,934 reads and 22,293 reads, respectively). In the case of ITS1 sequences, 26.48% (ctl) and 60.23% (vtl) of reads were classified and 20.29% (2851 reads, ctl) or 49.06% (11,272 reads, vtl) of them were matched to the kingdom Fungi, in total. Due to the high level of contamination of tested samples, mainly with genetic material of plant origin, the results of ITS1 analyses were additionally filtered. It allowed the analysis of only these taxonomic units which belong to the kingdom Fungi. Microorganisms belonging to the phyla Firmicutes, Proteobacteria, Ascomycota, and Basidiomycota were detected on both types of leather. Additionally, the chrome-tanned leathers were populated by bacteria of the phyla Actinobacteria and Bacteroidetes (relative abundance ≥ 2.00%). Bacteria belonging to the following families were predominant on particular types of leather: Enterobacteriaceae (relative abundance: 28.76%) and Paenibacillaceae (24.63%) on the ctl; Bacillaceae (33.16%), Oxalobacteraceae (23.80%), and Paenibacillaceae (23.31%) on the vtlThe predominant fungi belonged to the families Cystofilobasidiaceae (41.65%) and Mrakiaceae (43.94%) on the ctl and vtl, respectively. At all analyzed taxonomic levels, a greater variety of fungal taxa than bacterial were detected for all tested leathers. Table 3 shows all classifications at the species level with relative abundance ≥ 2.00% for at least one type of historical leather. On the surfaces of tested materials, bacterial operational taxonomic units (OTUs) with relative abundance < 2.00% assigned to the following genera were also detected: *Agrobacterium, Curtobacterium, Enhydrobacter, Erwinia, Janthinobacterium, Listeria, Lysinibacillus, Mycoplana, Novosphingobium, Oerskovia, Pedobacter, Psychrobacter, Rummeliibacillus, Solibacillus, Sporosarcina, Staphylococcus, Stenotrophomonas, Vagococcus*, and *Viridibacillus.* As regards fungi, the following OTUs with relative abundance < 2.00% were identified: *Alternaria, Botryotrichum, Botrytis, Candida, Chaetomium, Chrysosporium, Cladosporium, Cystobasidium, Daedaleopsis, Debaryomyces, Epicoccum, Fusarium, Gibberella, Microascus, Monocillium, Monodicyst, Mucor, Mycosphaerella, Myxotrichum, Neurospora, Pezicula, Pseudogymnoascus, Psora, Resinicum, Sporobolomyces, Trichoderma*, and *Wallemia*. Additionally, many of the OTUs did not match with cultivable microorganisms but showed 100% similarity along 100% length of sequences to various uncultured clones.

**TABLE 3:** Results of the identification of microorganisms detected on the spots on the historical leather shoes using culture-independent molecular methods.

Microbial taxa	Relative abundance of microorganisms [%]	
	Chrome-tanned leathers	Vegetable-tanned leathers
**Bacteria**		
*Acinetobacter lwoffii*	2.23	< 2.00
*Bacillus flexus*^x^	< 2.00	12.18
*Bacillus* unid.	5.02	18.41
*Carnobacterium funditum*^x^	< 2.00	6.66
*Chryseobacterium halperniae*^x^	5.01	0.00
*Moraxellaceae* unid.	7.98	0.00
*Oxalobacteriaceae* unid.^x^	< 2.00	23.70
*Pantoea agglomerans*	28.09	< 2.00
*Paenibacillus* unid.	24.62	23.29
*Planococcaceae* unid.^x^	9.39	2.67
*Pseudomonas* unid.^x^	< 2.00	3.98
*Rhodococcus* unid.^x^	5.55	< 2.00
**Fungi**		
*Aspergillus conicus*^x^	< 2.00	2.58
*Aspergillus versicolor*^x^	2.31	< 2.00
*Aureobasidium pullulans*	< 2.00	2.12
*Cystofilobasidium capitatum*^x^	41.65	0.00
*Filobasidium magnum*^x^	0.00	9.54
*Malasezzia restricta*^x^	6.03	2.80
*Mariannaea pinicola*^x^	4.35	< 2.00
*Myxotrichaceae* unid.^x^	2.42	< 2.00
*Naganishia diffluens*^x^	< 2.00	3.38
*Nectria ramulariae*^x^	13.71	6.72
*Penicillium thomii*^x^	6.26	< 2.00
*Selenophoma mahoniae*^x^	0.00	6.53
*Tausonia pullulans*^x^	0.00	43.89

**unid.–OTU identified to the level of order or family, unidentified to the genus level; ^*x*^–microbial taxa detected only using the culture-independent molecular methods.**

All microbial taxa detected using methods based on culturing were also identified as a result of NGS sequencing (with different relative abundances and at different levels of taxonomic identification). Moreover, molecular culture-independent methods proved the occurrence of additional bacterial and fungal taxa that were not detected by culture-dependent molecular methods (^x^ in Table 3).

A greater variety of bacteria (22 species) and a smaller variety of fungi (eight species) were found for chrome-tanned leathers than for vegetable-tanned ones (13 bacterial species and 10 fungal species) with the use of culture-dependent molecular methods. It was confirmed by results of metagenomic analyses. More bacterial OTUs were detected in the samples taken from ctl (59) than vtl (46) at the species level, and inversely in the case of fungal OTUs (64 and 115, respectively), regardless of relative abundance.

Microbial species that were isolated from tested spots on both types of historical leather with frequency > 50% for bacteria and > 30% for fungi, i.e., *A. lwoffii, B. cereus, B. subtilis, M. luteus, P. agglomerans, S. epideremidis, A. flavus, A. niger, C. cladosporioides, P. variotii*, and *P. chrysogenum*, were selected for further tests.

### Biocidal Effectiveness of Laser Irradiation in Various Variants

Results of tests of antimicrobial efficacy of laser irradiation at power of 0.3 W in CW mode with the use of two variants of exposure time and various numbers of repetitions against microorganisms inoculated on the model and historical leathers are presented in Tables 4, 5, respectively.

**TABLE 4:** Results of the biocidal effectiveness of laser irradiation in 0.3W/CW variant and various exposure times and number of repetitions on the model leather in regard to the unirradiated samples.

Microorganism	Type of sample				
	Unirradiated	Irradiated with 0.3 W/CW laser in various variants			
		1 × 30 s	2 × 30 s	1 × 2 min	2 × 2 min
	**Number of microorganisms [CFU/100 cm^2^]**				
*B. subtilis*	5.61 × 10^5^ ± 2.46 × 10^4^	3.29 × 10^5^ ± 1.35 × 10^4^	3.21 × 10^5^ ± 3.85 × 10^3^	2.54 × 10^5^ ± 1.17 × 10^4^	1.20 × 10^5^ ± 4.02 × 10^3^
*S. epidermidis*	4.19 × 10^5^ ± 1.02 × 10^4^	1.40 × 10^5^ ± 3.89 × 10^3^	1.10 × 10^5^ ± 1.90 × 10^3^	9.29 × 10^4^ ± 2.50 × 10^3^	5.60 × 10^4^ ± 1.53 × 10^3^
*A. niger*	6.94 × 10^5^ ± 2.27 × 10^4^	1.84 × 10^5^ ± 6.94 × 10^3^	1.50 × 10^5^ ± 3.85 × 10^2^	1.15 × 10^5^ ± 3.56 × 10^3^	5.91 × 10^4^ ± 1.92 × 10^2^
*A. flavus*	8.23 × 10^5^ ± 2.60 × 10^4^	2.36 × 10^5^ ± 6.94 × 10^3^	1.96 × 10^5^ ± 1.07 × 10^4^	1.92 × 10^5^ ± 5.09 × 10^3^	6.68 × 10^4^ ± 1.50 × 10^3^
	**Reduction [%]**				
*B. subtilis*	−	41.39*	42.77*	54.65*^$^	78.63*^#$^
*S. epidermidis*	−	66.68*	73.85*^#^	77.82*^$^	86.63*^#$^
*A. niger*	−	73.44*	78.43*^#^	83.38*^$^	91.49*^#$^
*A. flavus*	−	71.39*	76.25*^#^	76.65*^$^	91.89*^#$^

Number of microorganisms: mean ± standard deviation;Reduction: -not applied; * statistically significantly different to control (unirradiated) samples; ^#^statistically significant difference between single and double application of laser with the same exposure time; ^$^statistically significant difference between exposure times of 30 s and 2 min of laser application with the same number of repetitions; ^#^ and ^$^ symbols were placed with a greater reduction.

**TABLE 5:** Results of the biocidal effectiveness of laser irradiation in 0.3W/CW variant and various exposure times and number of repetitions on the historical leather in regard to the unirradiated samples.

Microorganism	Type of sample				
	Unirradiated	Irradiated with 0.3 W/CW laser in various variants			
		1 × 30 s	2 × 30 s	1 × 2 min	2 × 2 min
	**Number of microorganisms [CFU/100 cm^2^]**				
*B. subtilis*	6.00 × 10^5^ ± 3.51 × 10^4^	8.62 × 10^4^ ± 4.76 × 10^3^	9.93 × 10^4^ ± 6.17 × 10^3^	6.92 × 10^4^ ± 3.53 × 10^3^	8.89 × 10^1^ ± 1.92 × 10^1^
*B. subtilis* ATCC	5.34 × 10^5^ ± 3.85 × 10^3^	1.70 × 10^5^ ± 1.00 × 10^4^	1.53 × 10^5^ ± 2.08 × 10^3^	1.02 × 10^5^ ± 2.87 × 10^3^	3.96 × 10^4^ ± 1.35 × 10^3^
*A. lwoffii*	6.37 × 10^5^ ± 2.65 × 10^4^	2.49 × 10^5^ ± 1.26 × 10^4^	1.99 × 10^5^ ± 5.09 × 10^3^	1.80 × 10^5^ ± 8.82 × 10^3^	0.00 × 10^0^ ± 0.00 × 10^0^
*B. cereus*	3.72 × 10^5^ ± 1.02 × 10^4^	1.15 × 10^5^ ± 2.52 × 10^3^	3.36 × 10^4^ ± 2.46 × 10^3^	2.32 × 10^4^ ± 1.68 × 10^3^	1.43 × 10^4^ ± 2.27 × 10^2^
*M. luteus*	4.63 × 10^5^ ± 3.18 × 10^4^	2.11 × 10^5^ ± 1.26 × 10^4^	1.76 × 10^5^ ± 8.82 × 10^2^	8.71 × 10^4^ ± 2.22 × 10^3^	1.81 × 10^4^ ± 1.39 × 10^3^
*P. agglomerans*	6.81 × 10^5^ ± 3.85 × 10^3^	1.98 × 10^5^ ± 1.07 × 10^4^	2.50 × 10^5^ ± 1.45 × 10^4^	2.63 × 10^4^ ± 5.77 × 10^2^	1.06 × 10^4^ ± 1.35 × 10^2^
*S. epidermidis*	4.27 × 10^5^ ± 3.33 × 10^3^	4.00 × 10^2^ ± 8.82 × 10^1^	1.42 × 10^3^ ± 1.02 × 10^2^	1.00 × 10^2^ ± 0.00 × 10^0^	0.00 × 10^0^ ± 0.00 × 10^0^
*A. niger*	3.13 × 10^5^ ± 1.76 × 10^4^	3.81 × 10^3^ ± 1.58 × 10^2^	4.44 × 10^1^ ± 1.92 × 10^1^	0.00 × 10^0^ ± 0.00 × 10^0^	0.00 × 10^0^ ± 0.00 × 10^0^
*A. niger* ATCC	8.10 × 10^5^ ± 4.04 × 10^4^	1.88 × 10^4^ ± 1.90 × 10^3^	7.48 × 10^3^ ± 1.95 × 10^2^	0.00 × 10^0^ ± 0.00 × 10^0^	0.00 × 10^0^ ± 0.00 × 10^0^
*A. flavus*	3.03 × 10^5^ ± 1.20 × 10^4^	5.13 × 10^3^ ± 2.31 × 10^2^	3.33 × 10^1^ ± 3.33 × 10^1^	3.97 × 10^3^ ± 2.33 × 10^2^	0.00 × 10^0^ ± 0.00 × 10^0^
*C. cladosporioides*	9.02 × 10^5^ ± 3.15 × 10^4^	1.06 × 10^5^ ± 1.84 × 10^3^	6.96 × 10^4^ ± 1.84 × 10^3^	7.67 × 10^2^ ± 1.33 × 10^2^	0.00 × 10^0^ ± 0.00 × 10^0^
*P. variotii*	2.50 × 10^5^ ± 1.33 × 10^4^	8.69 × 10^4^ ± 2.50 × 10^3^	1.56 × 10^4^ ± 1.17 × 10^3^	6.11 × 10^2^ ± 1.02 × 10^2^	0.00 × 10^0^ ± 0.00 × 10^0^
*P. chrysogenum*	2.02 × 10^5^ ± 6.94 × 10^3^	4.69 × 10^4^ ± 8.39 × 10^2^	2.44 × 10^4^ ± 1.68 × 10^3^	3.56 × 10^2^ ± 3.85 × 10^1^	0.00 × 10^0^ ± 0.00 × 10^0^
	**Reduction [%]**				
*B. subtilis*	−	85.63*	83.44*	88.46*	99.99*^#$^
*B. subtilis* ATCC	−	68.19*	71.43*^#^	80.89*^$^	92.60*^#$^
*A. lwoffii*	−	60.91*	68.76*^#^	71.73*^$^	100.00*^#$^
*B. cereus*	−	69.01*	90.99*^#^	93.76*^$^	96.15*^#$^
*M. luteus*	−	54.44*	62.09*^#^	81.20*^$^	96.09*^#$^
*P. agglomerans*	−	70.96*^#^	63.30*	96.13*^$^	98.44*^#$^
*S. epidermidis*	−	99.91*	99.67*	99.98*	100.00*
*A. niger*	−	98.78*	99.99*	100.00*	100.00*
*A. niger* ATCC	−	97.68*	99.08*	100.00*	100.00*
*A. flavus*	−	98.31*	99.99*	98.69*	100.00*
*C. cladosporioides*	−	88.28*	92.29*^#^	99.92*^$^	100.00*^$^
*P. variotii*	−	65.24*	93.78*^#^	99.76*^$^	100.00*^$^
*P. chrysogenum*	−	76.81*	87.91*^#^	99.82*^$^	100.00*^$^

Number of microorganisms: mean ± standard deviation;Reduction: - not applied; * statistically significantly different to control (unirradiated) samples; ^#^statistically significant difference between single and double application of laser with the same exposure time; ^$^statistically significant difference between exposure times of 30 s and 2 min of laser application with the same number of repetitions; ^#^ and ^$^ symbols were placed with a greater reduction

Irradiation of samples of the model leather with the laser in four variants of operating parameters caused statistically significant differences in the number of all tested environmental strains in regard to unirradiated samples (^∗^ in Table 4). Double treatment gave a statistically significantly higher biocidal effect than a single application in most cases (^#^ in Table 4). This effect was also achieved for 2 min treatment in comparison with 30 s exposure for all microorganisms, regardless of the number of repetitions (^$^ in Table 4). The highest percentage reduction in the number of bacteria and fungi was obtained by irradiating samples of the model leather with the laser in the 0.3 W/CW 2 × 2 min variant.

The differences in the numbers of all microorganisms between unirradiated samples of the historical leather and irradiated with the laser in all tested variants of operating parameters were statistically significant (^∗^ in Table 5). A double application of the laser for 30 s gave a higher biocidal effect than a single application in seven out of 13 cases (^#^ in Table 5). With the use of one repetition, extending exposure time from 30 s to 2 min provided a statistically significantly higher percentage reduction in the number of most of the tested microbial strains (^$^ in Table 5) or complete elimination of microorganisms. The same relationship was observed for double treatment, but it accounted for all tested microorganisms. Laser irradiation in the 0.3 W/CW variant was the most effective with double 2 min application. It resulted in complete elimination of *A. lwoffii, S. epidermidis* (*R* = 5.80 log and *R* = 5.63 log), and all fungal strains (*R* > 5.30 log) and the highest reduction in the number of other tested bacterial strains (*R* = 1.13–3.83 log).

Regardless of the laser parameters used, bacteria of the species *S. epidermidis* (*R* = 2.48–5.63 log) and both strains of *A. niger* (environmental and from the collection of pure cultures, *R* = 1.63–5.91 log) were the most susceptible to disinfection on the historical leather, while *B. subtilis* ATCC, *B. cereus*, and *M. luteus* were the least sensitive (*R* ≤ 1.5 log).

### Effect of Laser Irradiation in Various Variants on the Optical Properties of the Model and Historical Leathers

The total color change (Δ*E*^∗^) following laser irradiation at power of 0.3 W and CW mode in four variants of exposure time and number of repetitions did not exceed 1.67 CIE*L^∗^a^∗^b^∗^* units for all tested leathers (Table 6). The values of Δ*E*^∗^ increased along with the increase of total time of exposure. The values of Δ*E*^∗^ were slightly higher for the model leathers than for the historical ones. Each tested variant of laser treatment caused a slight increase in lightness of the model leathers (Δ*L*^∗^ from 1.08 to 1.82). However, in the case of historical materials, only variants with 2 min exposure caused such alterations (Δ*L*^∗^ from 1.22 to 1.75). The values of Δ*a*^∗^ and Δ*b*^∗^ were not greater than ± 0.76 for the model leathers and ± 0.64 for the historical ones. After irradiation of the historical leathers with laser in the 1 × 30 s, 2 × 30 s, and 1 × 2 min variants the values of the total color change corresponding to no difference between two colors were obtained (Δ*E^∗^* < 0.87 CIE*L^∗^a^∗^b^∗^* units). Laser irradiation using the 0.3 W/CW 2 × 2 min variant did not result in a noticeable color change in the tested model or historical leathers (Δ*E^∗^* < 1.74 CIE*L^∗^a^∗^b^∗^* units).

**TABLE 6:** The total color changes in the model and historical leather after laser irradiation in 0.3 W/CW variant and various exposure times and number of repetitions

Type of material	Total color change Δ*E*^∗^ [CIE*L^∗^a^∗^b^∗^* units]			
	1 × 30 s	2 × 30 s	1 × 2 min	2 × 2 min
**Model leathers**				
Chrome-tanned	0.69	0.86	1.12	1.67
Vegetable-tanned	0.92	0.96	1.22	1.65
**Historical leathers**				
Chrome-tanned	0.43	0.50	0.78	1.62
Vegetable-tanned	0.23	0.25	0.75	1.13

The 0.3 W/CW 2 × 2 min variant of laser irradiation was the most biocidally effective against tested microorganisms and at the same time did not visibly change colors of the collagen-based materials.

### Effect of Laser Irradiation in the Selected Variant on the Physico-Chemical Properties of the Model and Historical Leathers

**FIGURE 2:**
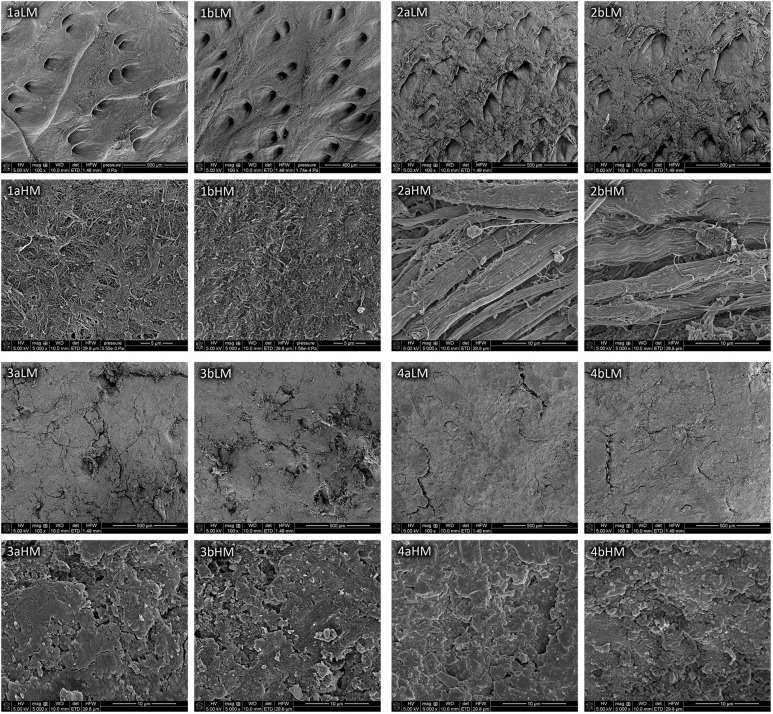
SEM images of the model chrome-tanned leather (1), model vegetable-tanned leather (2), historical chrome-tanned leather (3), and historical vegetable-tanned leather (4), unirradiated (a) and irradiated with laser in variant of 0.3 W/CW 2 × 2 min (b) taken at low magnification (LM) and high magnification (HM).

The morphology of samples of the model and historical leathers unirradiated and irradiated with a laser in a selected variant are illustrated by SEM micrographs (Figure 2). Comparison of the microscopic images in appropriate pairs (a and b) did not show any significant differences in morphology of the surfaces of all tested samples and appearance of collagen fibers from the model leathers resulting from laser treatment in the 0.3 W/CW 2 × 2 min variant. Unlike the model materials, SEM images of the historical leathers (3aHM and 4aHM) showed the presence of a solid layer of protective substances and impurities on the surfaces, which mask collagen fibers.

Analyses of obtained FTIR spectra showed that no significant changes in the structure of the model and historical leathers due to laser irradiation in the selected variant were detected (Supplementary Figure 1). As a consequence of the treatment, no new bands appeared and no bands disappeared in the whole spectral range. Collagen characteristic bands were predominant in the spectra of both tested model materials (1 and 2), while spectra of the model vegetable-tanned leather (2) also contained tannin absorption bands at 1,613, 780, and 1,314 cm^–1^ related to *ν*C = C stretching, *γ*C–H out of plane vibrations in aromatic rings, and *ν*C–O, *δ*O–H vibrations, respectively. Compared to samples of the model leathers, spectra of historical ones were characterized by intensive and broad bands between 1,200 and 850 cm^–1^ with the maximum at 1,007 cm^–1^, which may be attributed to *ν*(Si–O) and *ν*(C–O) stretching vibrations in the layer of protective substances and impurities covering the historical materials.

Parameters concerning bands representing amides I and II in the spectra of all tested leathers are summarized in Table 7. The obtained results indicate that laser irradiation did not cause degradation of the model and historical materials. The slight difference between positions of main peaks of the amide I and amide II (Δν = max. +2 cm^–1^) and between their intensity ratios (IA_I_/IA_II_ = max. ± 4) suggested that photooxidation or hydrolysis of both types of model leather and historical chrome-tanned leather certainly did not occur after laser irradiation in the 0.3 W/CW 2 × 2 min variant. In the case of historical vegetable-tanned leather, intensity of the amide I band increased by 0.18 with respect to amide II band after the treatment. However, the value of IA_I_/IA_II_ did not exceed 1.60 or 1.80, and no other symptoms which could suggest the occurrence of phootoxidation or hydrolysis were observed in the FTIR spectrum; therefore it can be concluded that these processes of degradation did not take place.

**TABLE 7:** Results of FTIR and XPS analyses of surfaces of the model and historical leathers unirradiated and irradiated with laser in variant of 0.3 W/CW 2 × 2 min.

Type of material	Type of sample (spectrum)	Parameters of degradation of leathers (FTIR analyses)				Parameters of elemental composition and condition of surfaces (XPS analyses)		
			
		νA_I_ [cm^–1^]	νA_II_ [cm^–1^]	Δν [cm^–1^]	IA_I_/A_II_	C:O	C_oxid_:C_unoxid_	O_carb_:O_other_
**Model leathers**								
Chrome-tanned	unirradiated (1a)	1,632	1,544	88	1.22	4.0:1.00	0.43:1.00	0.70:1.00
	irradiated (1b)	1,633	1,544	89	1.23	4.3:1.00	0.43:1.00	0.59:1.00
Vegetable-tanned	unirradiated (2a)	1,647	1,551	96	1.65	4.8:1.00	0.37:1.00	0.55:1.00
	irradiated (2b)	1,648	1,551	97	1.69	4.7:1.00	0.33:1.00	0.75:1.00
**Historical leathers**								
Chrome-tanned	unirradiated (3a)	1,647	1,545	102	1.27	2.7:1.00	0.33:1.00	0.32:1.00
	irradiated (3b)	1,647	1,543	104	1.26	2.5:1.00	0.31:1.00	0.36:1.00
Vegetable-tanned	unirradiated (4a)	1,647	1,545	102	1.26	5.5:1.00	0.31:1.00	0.51:1.00
	irradiated (4b)	1,647	1,544	103	1.44	6.1:1.00	0.15:1.00	0.54:1.00

**FTIR analyses: A_*I*_–amide I; A_*II*_–amide II; νA_*I*_–position of A_*I*_ main peak; νA_*II*_–position of A_*II*_ main peak; Δν–distance between positions of A_*I*_ and A_*II*_; IA_*I*_/A_*II*_–intensity ratio between A_*I*_ and A_*II*_ bands;XPS analyses: C:O–ratio of the number of carbon atoms to the number of oxygen atoms; C_*oxid*_:C_*unoxid*_–ratio of the sum of oxidized forms of carbon atoms to the sum of unoxidized (aliphatic and aromatic) forms of carbon (degree of oxidation); O_*carb*_:O_*other*_—ratio of the sum of carbonyl groups to the sum of other oxygen-containing forms of carbon (surface polarity).**

Analyses of the XPS spectra of unirradiated samples of the model and historical leathers in comparison with the spectra of irradiated ones showed no significant effect of laser treatment on the chemical properties of the tested materials (Supplementary Figure 2). The elemental compositions of individual materials are presented in Supplementary Tables 1, 2. Carbon, oxygen, and nitrogen were predominant in all samples. Additionally, elements such as S, Si, N, Ca, Na, Al, Mg, and Zn in small amounts and varying proportions were observed in samples of the historical leathers. This confirms the presence of a secondary layer on surfaces of the historical materials. No signals from Cr were found in a survey spectra of either type of chrome-tanned leather. It indicates that chromium is not contained in the outermost surface of these materials but remains in deeper layers. Moreover, trace amounts of water were also found in the samples of the model and historical chrome-tanned leathers.

The distribution of deconvolution peaks derived from the XPS narrow spectra of C1s and O1s regions are shown in Supplementary Table 3 and results of spectral analyses are briefly summarized in Table 7. Laser in the 0.3 W/CW 2 × 2 min variant did not interact appreciably with the outermost surface of the model and historical leathers. From the tabular data it follows that C:O ratios on the surface of the leathers changed rather randomly after laser treatment, and the maximum difference between unirradiated and irradiated samples was ± 0.6. No significant variations in degree of surface oxidation between untreated and laser-treated samples of both model leathers and historical chrome-tanned leather were observed. The subtle differences (max. ± 0.04) in the C_oxid_:C_unoxid_ ratio for these leathers were within the margin of error, while the difference for samples 4a and 4b was greater (± 0.16). SEM micrographs (Figure 2) and data in Supplementary Table 2 show that surfaces of samples of the historical vegetable-tanned leather are highly non-homogeneous due to the presence of impurities and protective substances. It might cause instability of the results of the XPS analyses. Therefore, greater differences in the above-mentioned ratios for this type of leather than for other ones may be partly owing to the surface heterogeneity and partly to a slight decrease in the content of oxygen and oxidized forms of carbon in the layer covering the historical material after laser irradiation.

Comparison of O_carb_:O_other_ ratios for corresponding pairs of samples of the model leathers indicated a changeable trend, i.e., a slight decrease or increase in surface polarity of chrome- and vegetable-tanned leathers as a result of laser irradiation, respectively. However, results obtained for sample 1b are inconsistent with the results of FTIR analyses. Changes in the O_carb_:O_other_ ratio for the model leathers (max. ± 0.20) were greater than these for historical ones (max. +0.04), for which no significant effect of laser irradiation on surface polarity was observed.

## Discussion

At the A-BSM, maintaining proper microbiological cleanliness of all historical objects containing degraded materials is a prerequisite to ensure their appropriate condition. Conducting microbiological tests before conservation treatments and application of suitable methods of disinfection are crucial for the preservation of objects against biodeterioration and are necessary to protect the health of employees of the museum.

In the first stage of the study, small spots observed on the surfaces of 30 historical leather shoes stored in the collection of the A-BSM were subjected to assessment of microbiological contamination. A similar number of fungi and a larger number of bacteria were found on the spots on the degraded collagenous materials on tested shoes than on the fabric, cardboard, and wooden elements on shoes from the museum (Rybitwa et al., 2020; Wawrzyk et al., 2020). The average concentration of bacteria obtained in this study was lower, and the one of fungi was similar to that of microorganisms inhabiting leather shoes of prisoners from the collection of the State Museum at Majdanek, Poland, which also originate from the period of World War II (Perkowski and Goździecki, 2002; Falkiewicz-Dulik, 2015).

There are not many new scientific reports describing microbiological analyses of historical leather objects. Most of them are related to other collagen-based materials such as parchment. Strzelczyk and Karbowska-Berent (2004) and Strzelczyk (2004) stated that parchment and leather are inhabited by a very similar microbiota, therefore results of the current study were partially compared to tests on historical objects made of parchment. Many culturable bacterial and fungal species inhabiting leather shoes from the A-BSM, especially these belonging to the following genera–*Acinetobacter, Bacillus, Micrococcus, Paenibacillus, Staphylococcus, Aspergillus, Aureobasidium, Cladosporium*, and *Penicillium*–were frequently isolated from historical parchment documents (Kraková et al., 2012; Gutarowska, 2016; Lech, 2016; Paiva de Carvalho et al., 2016). Apart from parchment-specific microorganisms, fungi of the genus *Paecilomyces* usually occur on surfaces of historical objects made of leather (Strzelczyk, 2004). Their presence was confirmed in this study as well. All fungi detected on surfaces of tested shoes by culture-dependent methods, including *G. candidum, H. verticilata*, and *P. variotii*, were also found in the environments of archives and tanneries (Pinheiro et al., 2011; Pinheiro, 2014; Skóra et al., 2014). Bacteria isolated from the leather shoes belong to the same genera as these contaminating various historical cellulosic movable objects at the A-BSM (Wawrzyk et al., 2018, 2020; Rybitwa et al., 2020). Most of the microorganisms detected in the current studies using culture-independent methods (with relative abundance ≥ 2.00% and some fungi with lesser one, such as *Alternaria, Botrytis, Chaetomium, Epicoccum, Fusarium, Mucor*, and *Trichoderma*) were also previously detected on collagenous materials in many studies with the use of clone libraries construction, PCR-denaturing gradient gel electrophoresis, and microscopic methods (Troiano et al., 2014; Piñar et al., 2015; Lech, 2016; Karakasidou et al., 2018; Liu et al., 2018). NGS sequencing is one of the most commonly utilized methods that is not based on cultivation to study microorganisms involved in biodeterioration of historical buildings (Adamiak et al., 2017; Huang et al., 2017; Liu et al., 2017) and book collections (Gutarowska, 2016; Kraková et al., 2018). However, in the case of parchment documents and leather objects, this method was used only by one group of researchers (Migliore et al., 2017, 2019; Perini et al., 2019). The subjects of their analyses were purple spot damages of parchment and commercial leather, triggered by haloarchaea, but including several other microbial species succeeding with time and not other kinds of damages as done in this study. The application of metagenomic analyses allowed for the detection of the following microorganisms on the surfaces of tested leather shoes from the A-BSM, the identification of which was not possible using culture-dependent methods: *Bacillus flexus, Carnobacterium funditum, Chryseobacterium halperniae, Pseudomonas* spp., *Rhodococcus* spp., *Aspergillus conicus, Aspergillus versicolor, Cystofilobasidium capitatum, Filobasidium magnum, Malasezzia restricta, Mariannaea pinicola, Naganishia diffluens, Nectria ramulariae, Penicillium thomii, Selenophoma mahoniae*, and *Tausonia pullulans* (relative abundance ≥ 2.00%). The culture-independent molecular methods ensured detection of more fungal taxa than culture-based molecular ones, which is consistent with results of analyses carried out by Wu et al. (2019).

Leather contains protein, lipids, and tannins, and thus provide a suitable substrate for the development of many microorganisms. According to literature, most of the microorganisms isolated from tested shoes show leather-biodeteriorative potential. Leather damage is mostly caused by fungi, and by bacteria to be a lesser extent, and may be manifested as discolorations, colored stains, mold stains, depressions, and weakness of strength (Orlita, 2004; IAEA, 2017). Biodeterioration of historical leather may result from the decomposition of fats introduced into the material during conservation treatments, carried out by *Aureobasidium pullulans*, *Paecilomyces* spp., and bacteria possessing lipolytic properties such as *P. agglomerans*. As a result, fatty acids are formed. They may migrate to the surface and appear as white salty deposits or react with tannins and cause discolorations (Strzelczyk and Karbowska-Berent, 2004; Zhen-Qian and Chun-Yan, 2009). Stains may also appear due to the activity of tannase produced by *Aspergillus* and *Penicillium* (Falkiewicz-Dulik, 2015). Microorganisms showing proteolytic properties are also undesirable on historical objects, especially *A. lwoffii, B. cereus, B. megaterium, B. subtilis, M. luteus, S. epidermidis, A. flavus, A. versicolor, C. cladosporioides, P. variotii, P. chrysogenum*, and various species of fungi belonging to the genera *Alternaria, Aspergillus, Aureobasidium, Chaetomium, Epicoccum, Fusarium, Mucor, Penicillium*, and *Trichoderma* (Strzelczyk, 2004; Mitchell and McNamara, 2010; Kraková et al., 2012). It is assumed that chrome-tanned leather is more resistant to fungal attack than vegetable-tanned leather owing to the presence of chromium oxide. However, most historical leather objects are regularly greased, therefore the difference between the conditions for development of microorganisms is reduced (Falkiewicz-Dulik, 2015; IAEA, 2017), which was also observed in this study for leather shoes.

Among the microorganisms isolated from the A-BSM shoes, species that may be harmful to the health of people who carry out conservation treatments and inspect the historical collection by causing allergic reactions, poisonings, organ infections, and mycotoxicosis were found. *A. lwoffii* can cause gastroenteritis and wound infections (Rathinavelu et al., 2003). *P. agglomerans* may be a causative agent of infections of wounds resulting from piercing or laceration of the skin (Dutkiewicz et al., 2016). *B. cereus* may be an etiological factor of serious intestinal or non-gastrointestinal-tract infections, and less often of eye infections (Kamar et al., 2013). *S. epidermidis* is a pathogen involved in subacute and chronic infections of the eye, ear, nose, throat, endophthalmitis, and cardiovascular infections (Vuong and Otto, 2002). *Alternaria, Aspergillus, Cladosporium*, and *Penicillium* may cause allergies. Fungal infections of internal and external organs can be caused, among others, by *Aspergillus* (bronchopulmonary and invasive aspergillosis), *Candida* (superficial and cutaneous candidiasis), *Mucor* (pulmonary and naso-orbito-cerebral mucormycosis), and *Penicillium* (pulmonary penicillosis) (Levinson, 2016). Mycotoxins are characterized by carcinogenic, mutagenic, and teratogenic effects, and can cause both acute and chronic food poisoning. They are produced by fungi such as *Aspergillus* spp. (*A. flavus*–aflatoxins and cyclopiazonic acid, *A. niger*–ochratoxin), *Penicillium* spp. (citrinin, cyclopiazonic acid, ochratoxin and patulin), and *Fusarium* (zearalenone and fumonisins) (Bennett and Klich, 2003; Pinheiro, 2014). Some of the above-mentioned microorganisms may occur on human skin or in the environment and are not obligate infectious agents. However, under adverse conditions, they may become infectious agents for immunocompromised people.

The microbiological assessment of small spots on the leather shoes revealed the presence of microorganisms showing biodeteriorative potential and posing a threat to human health, therefore they were qualified for disinfection. Due to the small size of microbiologically contaminated areas, the possibility of using laser irradiation to eliminate harmful microorganisms was considered. Consequently, in the next stage of the study the values of which diode laser operating parameters would be the most biocidally effective and would not cause visual alterations in the model (new) and historical (degraded) leathers was checked. These criteria were met for double exposure for 2 min (0.3 W/CW 2 × 2 min), which resulted in a 96.09–100.00% reduction in the number of individual bacterial strains and complete elimination of all tested fungi. However, a greater sensitivity to the disinfection of fungi than bacteria was demonstrated and a higher biocidal effect on historical material than on model material was achieved regardless of the laser parameters used. These results are satisfactory, because in conservation of cultural heritage it is assumed that fungi are more harmful to objects than bacteria (Strzelczyk, 2004). Model leather was used in this study only to check the effectiveness and effect of laser irradiation on the new material, which is not covered by protective substances and is not common at the museum. Additionally, for two strains from the collection of pure cultures, a slightly lower percentage reduction was obtained than for the corresponding species of environmental strains. The different susceptibility of environmental microorganisms to the disinfection may follow from their long-term contact with various biocidal, cleansing, and protective agents applied on the surfaces of historical leather shoes during conservation treatments. Previous studies on the antimicrobial effects of diode laser irradiation were mainly conducted in medicine and were focused on pathogenic bacteria such as *Enterococcus faecalis* and *Escherichia coli*. Nevertheless, higher values of average laser power than in this paper were most often applied (Beer et al., 2011; Asnaashari et al., 2016; Olivi et al., 2016; Dai et al., 2018), with similar ones applied less often (Tosun et al., 2012; Sadık et al., 2013).

The biocidal effectiveness of laser irradiation in the 0.3 W/CW 2 × 2 min variant obtained under laboratory conditions is similar to or higher than that of other disinfection methods used so far in the field of conservation of leather and parchment, e.g., gamma irradiation, fumigation with ethylene oxide (EtO), p-chloro-m-cresole (PCMC), and methods tested to apply to collagen-based cultural heritage objects (X-ray irradiation and misting with silver nanoparticles (AgNPs)). It is assumed that fungi die after gamma irradiation at a dose below 10 kGy, while bacteria, even the most resistant, at a dose up to 25 kGy regardless of the type of material (Cortella et al., 2011). Although, in the State Museum at Majdanek, an absorbed dose of 20 kGy was used to disinfect 60,000 historical leather shoes, and a reduction in the number of bacteria in the range of 95.0–99.9% and 80–97% for fungi was achieved (IAEA, 2017). Zerek (2014) showed that PCMC allowed for elimination of only some fungal strains tested by him, while EtO was absolutely effective against all fungi. However, in some cases, to achieve this effect the process needed to be repeated up to three times. Recently, the biocidal effectiveness of X-ray irradiation was tested on collagen-based materials for which a dose of 1 kGy gave satisfactory results (Vadrucci et al., 2019, 2020). Misting with AgNPs (8 cycles, 90 ppm) did not give as effective results as laser irradiation, because no statistically significant reduction in the number of microorganisms was demonstrated for more than half of the strains (Gutarowska et al., 2012, 2014).

A very important criterion applied by conservators is to show that the disinfection method to be used does not adversely affect the esthetic and functional properties of the materials. Therefore, assessment of color change was also used to choose an appropriate variant of laser operating parameters, and in the last stage, impact of laser irradiation in the selected variant on morphology, structure, elemental composition, and chemical condition of tested leathers was determined. An essential advantage of the applied methods of optical and physico-chemical analyses was the possibility to test samples of very small sizes (SEM and XPS) or the ability to not test them at all (color, FTIR), thereby protecting the historical objects (Možina et al., 2007). Due to this, noninvasiveness, color measurement, and FTIR-ATR spectrophotometry are frequently used techniques to investigate alterations in the appearance and structure of historical materials after various conservation treatments, including collagenous materials (Gutarowska et al., 2012; Vyskočilová et al., 2019; Vadrucci et al., 2020). SEM is sometimes used in this area to assess morphological modifications as well (Vadrucci et al., 2020). However, the utilization of XPS has not yet been demonstrated in scientific reports on studies of historical materials. This technique is very valuable, because it allows for the determination of variations in the outermost layer, as shown in this study. Laser treatment in the 0.3 W/CW 2 × 2 min variant did not cause a noticeable color change in the model and historical leathers. The same effect was obtained by Nunes et al. (2012) and Gutarowska et al. (2014) after applying gamma irradiation and misting with AgNPs to disinfect cultural heritage objects made of collagen-based materials. Tested historical leathers are covered with a layer of various substances, in contrast to the model materials, which is visible in SEM photographs. Owing to this, the structural and chemical properties of the leathers are different, what was confirmed by results of FTIR and XPS analyses. The properties of historical materials result from natural process of degradation under the influence of environmental factors and conservation treatments. Moreover, the surfaces of historical objects are coated with protective substances, the composition of which is not known in each case. These substances, together with impurities, constitute a barrier between the layer containing collagen, which has historical value, and external factors. Therefore, any significant changes in the surface morphology, structure of collagen, and surface polarity of the outermost layers of tested historical materials were detected after 1 month of laser irradiation. The different effects of laser treatment on C:O ratio and degree of oxidation were dependent on the tanning method of the historical leathers, although the results were not fully unequivocal.

The impact of laser irradiation on the collagen-based materials obtained in this study is comparable to or less harmful than that of other methods used and tested in the field of conservation. Gamma irradiation at a dose up to 10 kGy is safe for collagenous materials, while doses equal to or greater than 25 kGy cause structural modifications in the form of cross-links (Lungu et al., 2014; Sendrea et al., 2015, 2017). EtO reacts with proteins contained in leather and parchment making these materials harder and more brittle (Unger et al., 2001). X-ray irradiation at a dose of 1 kGy causes disorders in structure of collagen fibers due to the effects of a partial unwinding, what leads to gelatinization of historical leather. This is accompanied by a significant decrease in intensity of the amide I band with respect to amide II band (0.53), what is ascribable to hydrolysis of collagen (Vadrucci et al., 2020). Misting with AgNPs causes an 18% increase in elongation at the break of model leather (Gutarowska et al., 2014).

Moreover, in contrast to EtO and PCMC, which accumulate in disinfected objects and are then released into the environment, which exposes conservators to poisonous chemicals (Unger et al., 2001; Cortella et al., 2011; Zerek, 2014), the use of laser treatment does not create such negative effects.

The disinfection technique based on laser irradiation may also be suitable for other natural degraded materials. It has already been tested with good results on cellulosic materials (Rybitwa et al., 2020). However, the research presented in this paper did not include objects much older than 80–100 years, i.e., from the period of World War II. Application of biocidal agents always requires caution; therefore new disinfection techniques should always be tested on a limited number of objects and then their condition should be monitored. It might be applied to a larger group only after obtaining satisfactory results on this test group.

## Conclusion

The implementation of this multidisciplinary approach concluded that double 2 min irradiation with a medical diode laser at power of 0.3 W in continuous wave mode is suitable for decontaminating very small, microbiologically contaminated spots occurring on the surfaces of degraded collagenous materials. The selected variant of laser treatment completely eliminates all tested fungal strains from historical leather and reduces the number of bacteria by over 96%. This method does not result in a deterioration of color and physico-chemical properties of degraded historical leathers, which was confirmed by SEM and FTIR analyses. The subtle changes in these materials detected by XPS examination did not directly affect historical leather but were related only to the outermost non-historical layer, i.e., protective substances and impurities, which are usually removed during conservation treatments. The high biocidal effectiveness and lack of unacceptable changes in properties of the leather indicate that laser irradiation can be used for disinfecting historical leather objects.

## Data Availability Statement

The original contributions presented in the study are publicly available. This data can be found here: Sequence Read Archive (SRA) under BioProject ID: PRJNA676029.

## Author Contributions

DR, AW, and MR contributed to the conception and design of the study and wrote sections of the manuscript. DR was involved in acquisition of all data and performed statistical analyses, wrote the first draft of the manuscript, and took the lead in writing the manuscript. DR and AW analyzed and interpreted results of microbiological and physico-chemical tests. DR and MR interpreted results of tests of biocidal effectiveness of laser irradiation. AW and MR revised the manuscript critically for important intellectual content. All authors contributed to manuscript revision, read, approved the submitted version, and agree to be accountable for all aspects of the work.

## Conflict of Interest

The authors declare that the research was conducted in the absence of any commercial or financial relationships that could be construed as a potential conflict of interest.
