# Corrigendum: Application of a Medical Diode Laser (810 nm) for Disinfecting Small Microbiologically Contaminated Spots on Degraded Collagenous Materials for Improved Biosafety in Objects of Exceptional Historical Value From the Auschwitz-Birkenau State Museum and Protection of Human Health

**DOI:** 10.3389/fmicb.2021.673867

**Published:** 2021-03-22

**Authors:** Dorota Rybitwa, Anna Wawrzyk, Mansur Rahnama

**Affiliations:** ^1^Preservation Department, Auschwitz-Birkenau State Museum, Oświecim, Poland; ^2^Sanitary-Epidemiological Station, Kraków, Poland; ^3^The Chair and Department of Oral Surgery, Medical University of Lublin, Lublin, Poland

**Keywords:** diode laser, microorganisms, disinfection, biodeterioration, human health, collagenous materials, historical leather shoes, Auschwitz-Birkenau State Museum

In the original article, there was an error. We incorrectly stated that “…in the case of parchment documents and leather objects, this method was used only by one group of researchers (Migliore et al., 2017, 2019; Perini et al., 2019). The subject of their analyses was only haloarcheae, not the entire community of microorganisms inhabiting historical collagen-based materials as in this study.”

However, in all the cited papers the entire microbial communities are clearly shown, and the entire databases are deposited for use by the entire scientific community.

A correction has been made to the **Discussion**, paragraph three:

“There are not many new scientific reports describing microbiological analyses of historical leather objects. Most of them are related to other collagen-based materials such as parchment. Strzelczyk and Karbowska-Berent (2004) and Strzelczyk (2004) stated that parchment and leather are inhabited by a very similar microbiota, therefore results of the current study were partially compared to tests on historical objects made of parchment. Many culturable bacterial and fungal species inhabiting leather shoes from the A-BSM, especially these belonging to the following genera–*Acinetobacter, Bacillus, Micrococcus, Paenibacillus, Staphylococcus, Aspergillus, Aureobasidium, Cladosporium*, and *Penicillium*–were frequently isolated from historical parchment documents (Kraková et al., 2012; Gutarowska, 2016; Lech, 2016; Paiva de Carvalho et al., 2016). Apart from parchment-specific microorganisms, fungi of the genus *Paecilomyces* usually occur on surfaces of historical objects made of leather (Strzelczyk, 2004). Their presence was confirmed in this study as well. All fungi detected on surfaces of tested shoes by culture-dependent methods, including *G. candidum, H. verticilata*, and *P. variotii*, were also found in the environments of archives and tanneries (Pinheiro et al., 2011; Pinheiro, 2014; Skóra et al., 2014). Bacteria isolated from the leather shoes belong to the same genera as these contaminating various historical cellulosic movable objects at the A-BSM (Wawrzyk et al., 2018, 2020; Rybitwa et al., 2020). Most of the microorganisms detected in the current studies using culture-independent methods (with relative abundance ≥ 2.00% and some fungi with lesser one, such as *Alternaria, Botrytis, Chaetomium, Epicoccum, Fusarium, Mucor*, and *Trichoderma*) were also previously detected on collagenous materials in many studies with the use of clone libraries construction, PCR-denaturing gradient gel electrophoresis, and microscopic methods (Troiano et al., 2014; Piñar et al., 2015; Lech, 2016; Karakasidou et al., 2018; Liu et al., 2018). NGS sequencing is one of the most commonly utilized methods that is not based on cultivation to study microorganisms involved in biodeterioration of historical buildings (Adamiak et al., 2017; Huang et al., 2017; Liu et al., 2017) and book collections (Gutarowska, 2016; Kraková et al., 2018). However, in the case of parchment documents and leather objects, this method was used only by one group of researchers (Migliore et al., 2017, 2019; Perini et al., 2019). The subjects of their analyses were purple spot damages of parchment and commercial leather, triggered by haloarchaea, but including several other microbial species succeeding with time and not other kinds of damages as done in this study. The application of metagenomic analyses allowed for the detection of the following microorganisms on the surfaces of tested leather shoes from the A-BSM, the identification of which was not possible using culture-dependent methods: *Bacillus flexus, Carnobacterium funditum, Chryseobacterium halperniae, Pseudomonas* spp., *Rhodococcus* spp., *Aspergillus conicus, Aspergillus versicolor, Cystofilobasidium capitatum, Filobasidium magnum, Malasezzia restricta, Mariannaea pinicola, Naganishia diffluens, Nectria ramulariae, Penicillium thomii, Selenophoma mahoniae*, and *Tausonia pullulans* (relative abundance ≥ 2.00%). The culture-independent molecular methods ensured detection of more fungal taxa than culture-based molecular ones, which is consistent with results of analyses carried out by Wu et al. (2019).”

The authors apologize for this error and state that this does not change the scientific conclusions of the article in any way. The original article has been updated.
