# Supplementary material for: Application of a Medical Diode Laser (810 nm) for Disinfecting Small Microbiologically Contaminated Spots on Degraded Collagenous Materials for Improved Biosafety in Objects of Exceptional Historical Value From the Auschwitz-Birkenau State Museum and Protection of Human Health
Source: Front Microbiol. 2020 Dec 18;11:596852. doi: 10.3389/fmicb.2020.596852 (PMC7775414; doi:10.3389/fmicb.2020.596852)
Supplement: Supplementary file 1 [file Data_Sheet_1.docx]

Supplementary Material

# Supplementary Figures and Tables

## Supplementary Figures


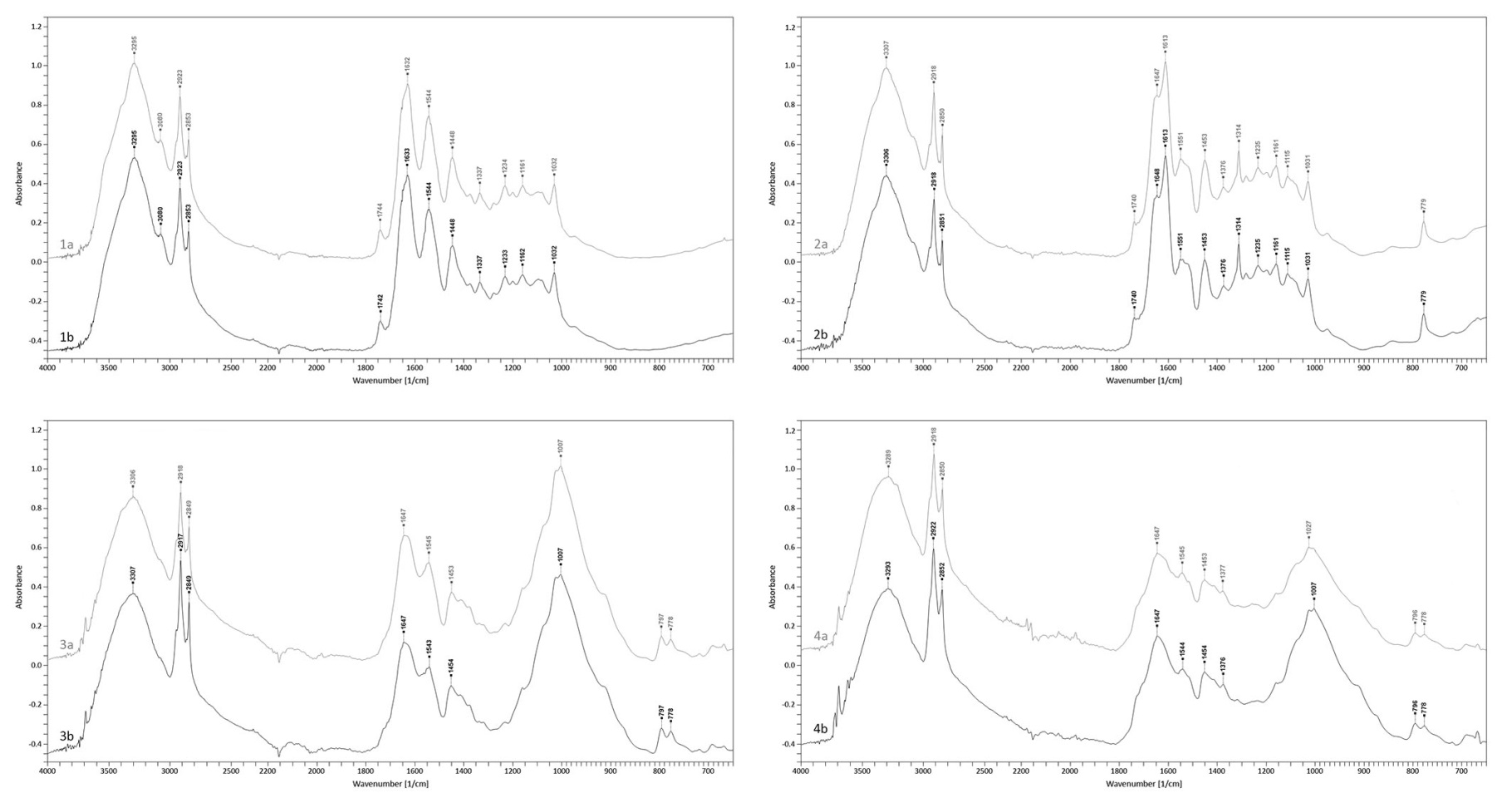


**Supplementary Figure 1.** FTIR spectra of the model chrome-tanned leather **(1)**, model vegetable-tanned leather **(2)**, historical chrome-tanned leather **(3)** and historical vegetable-tanned leather **(4)**, unirradiated **(a)** and irradiated with laser in variant of 0.3W/CW 2×2min **(b)**

**
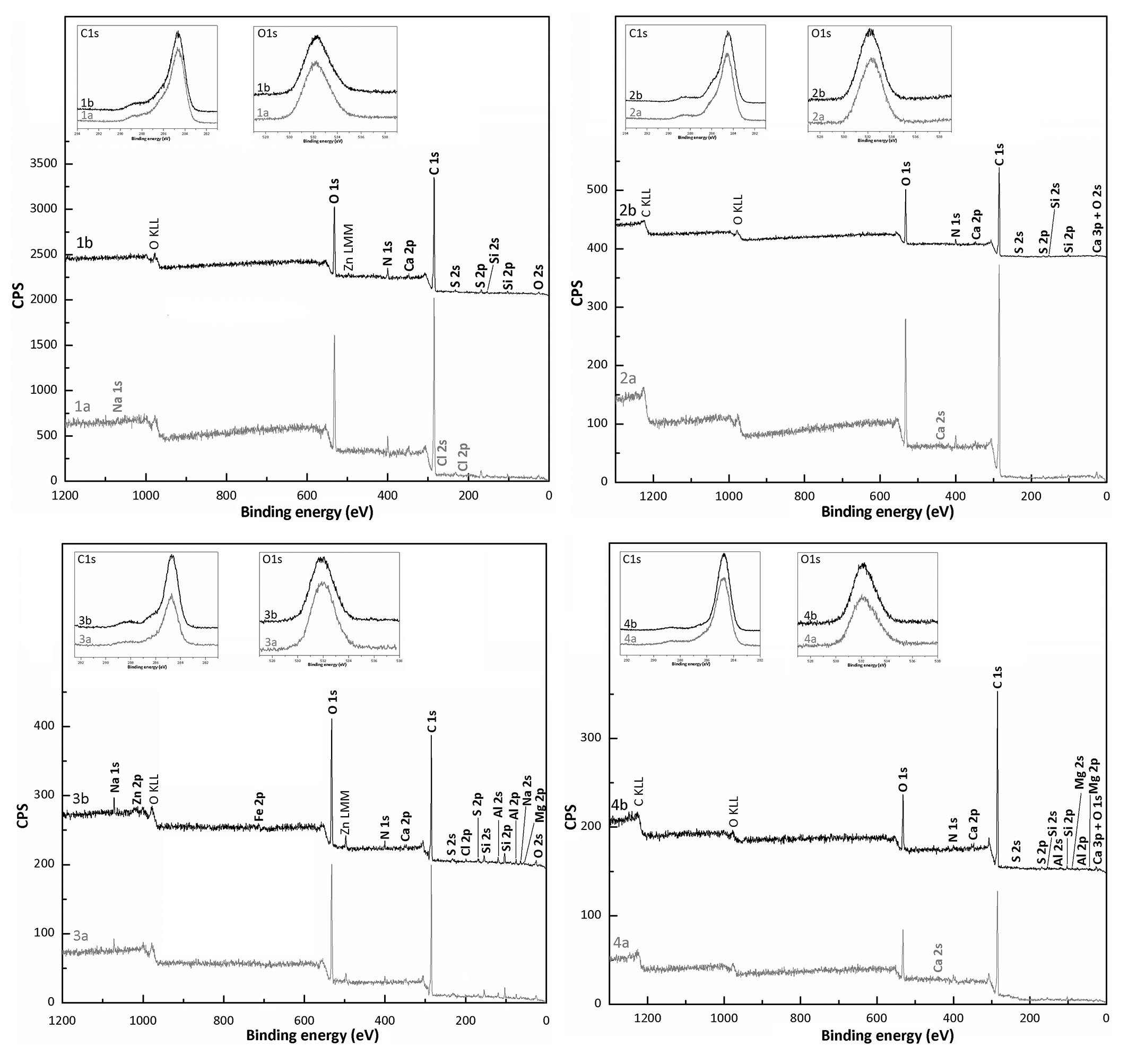
**

**Supplementary Figure 2.** XPS survey and regional (C1s and O1s) spectra of the model chrome-tanned leather **(1)**, model vegetable-tanned leather **(2)**, historical chrome-tanned leather **(3)** and historical vegetable-tanned leather **(4)**, unirradiated **(a)** and irradiated with laser in variant of 0.3W/CW 2×2min **(b)**

## Supplementary Tables

**Supplementary Table 1.** The elemental composition of surfaces of the model leathers unirradiated and irradiated with laser in variant of 0.3W/CW 2×2min determined by XPS method

| Spectral  band | Model chrome-tanned leather | | | | | | Model vegetable-tanned leather | | | | | |
| --- | --- | --- | --- | --- | --- | --- | --- | --- | --- | --- | --- | --- |
|  | unirradiated (1a) | | | irradiated (1b) | | | unirradiated (2a) | | | irradiated (2b) | | |
|  | E_B_ [eV] | % At | % Mass | E_B_ [eV] | % At | % Mass | E_B_ [eV] | % At | % Mass | E_B_ [eV] | % At | % Mass |
| Si 2p | 102.78 | 1.0 | 2.1 | 101.81 | 2.3 | 4.7 | 101.70 | 1.1 | 2.4 | 102.20 | 2.0 | 4.2 |
| S 2p | 168.28 | 2.3 | 5.3 | 168.31 | 1.9 | 4.3 | 167.70 | 0.5 | 1.2 | 168.70 | 0.7 | 1.6 |
| Cl 2p | 198.78 | 0.2 | 0.5 | - | - | - | - | - | - |  |  |  |
| C 1s | 284.78 | 72.7 | 63.1 | 284.81 | 73.6 | 64.2 | 284.70 | 79.3 | 72.5 | 284.70 | 78.1 | 70.4 |
| Ca 2p | 346.78 | 1.1 | 3.2 | 347.31 | 0.9 | 2.5 | 348.20 | 0.6 | 1.7 | 347.20 | 0.5 | 1.5 |
| N 1s | 399.78 | 4.3 | 4.4 | 399.81 | 4.3 | 4.3 | 399.70 | 1.9 | 2.0 | 400.20 | 2.2 | 2.3 |
| O 1s | 531.78 | 18.0 | 20.8 | 531.81 | 17.2 | 20.0 | 532.20 | 16.6 | 20.2 | 532.20 | 16.6 | 20.0 |
| Na 1s | 1068.28 | 0.4 | 0.6 | - | - | - | - | - | - | - | - | - |

E_B_ - binding energy; % At - atomic percentage; % Mass - mass percentage

**Supplementary Table 2.** The elemental composition of surfaces of the historical leathers unirradiated and irradiated with laser in variant of 0.3W/CW 2×2min determined by XPS method

| Spectral  band | Historical chrome-tanned leather | | | | | | Historical vegetable-tanned leather | | | | | |
| --- | --- | --- | --- | --- | --- | --- | --- | --- | --- | --- | --- | --- |
|  | unirradiated (3a) | | | irradiated (3b) | | | unirradiated (4a) | | | irradiated (4b) | | |
|  | E_B_ [eV] | % At | % Mass | E_B_ [eV] | % At | % Mass | E_B_ [eV] | % At | % Mass | E_B_ [eV] | % At | % Mass |
| Mg 2p/2s | 56.25 | 1.3 | 2.1 | 56.37 | 2.2 | 3.3 | - | - | - | 51.20 | 2.5 | 4.4 |
| Al 2p | 73.75 | 3.9 | 7.0 | 74.87 | 4.0 | 6.9 | - | - | - | 76.20 | 2.0 | 3.8 |
| Si 2p | 102.25 | 6.1 | 11.3 | 102.87 | 6.1 | 10.9 | 102.19 | 3.0 | 6.2 | 102.70 | 2.2 | 4.6 |
| S 2p | 168.25 | 0.8 | 1.6 | 167.37 | 0.8 | 1.6 | 169.69 | 1.1 | 2.6 | 169.20 | 0.5 | 1.3 |
| Cl 2p | 200.25 | 0.3 | 0.6 | 199.37 | 0.3 | 0.8 | - | - | - | - | - | - |
| C 1s | 284.75 | 61.9 | 49.1 | 284.87 | 58.7 | 44.4 | 284.69 | 77.2 | 68.0 | 284.70 | 79.8 | 69.5 |
| Ca 2p | 347.25 | 0.7 | 1.8 | 346.87 | 0.7 | 1.9 | 354.69 | 1.1 | 3.1 | 346.20 | 0.9 | 2.5 |
| N 1s | 399.75 | 1.6 | 1.5 | 399.87 | 1.9 | 1.7 | 400.19 | 3.7 | 3.8 | 399.70 | 1.1 | 1.1 |
| O 1s | 531.75 | 22.9 | 24.1 | 531.87 | 23.5 | 23.6 | 531.69 | 14.0 | 16.4 | 532.20 | 11.1 | 12.8 |
| Fe 2p | - | - | - | 713.37 | 0.5 | 1.8 | - | - | - | - | - | - |
| Zn 2p | - | - | - | 1012.87 | 0.5 | 2.1 | - | - | - | - | - | - |
| Na 1s | 1071.75 | 0.6 | 1.0 | 1071.37 | 0.8 | 1.2 | - | - | - | - | - | - |

E_B_ - binding energy; % At - atomic percentage; % Mass - mass percentage

**Supplementary Table 3.** Results of curve-fitting of high resolution XPS spectra for the C1s and O1s regions in the model and historical leathers unirradiated and irradiated with laser in variant of 0.3W/CW 2×2min

| Spectral band | Chemical group | Model chrome-tanned leather | | | | Model vegetable-tanned leather | | | | Historical chrome-tanned leather | | | | Historical vegetable-tanned leather | | | |
| --- | --- | --- | --- | --- | --- | --- | --- | --- | --- | --- | --- | --- | --- | --- | --- | --- | --- |
|  |  | unirradiated (1a) | | irradiated (1b) | | unirradiated (2a) | | irradiated (2b) | | unirradiated (3a) | | irradiated (3b) | | unirradiated (4a) | | irradiated (4b) | |
|  |  | E_B_ [eV] | % At | E_B_ [eV] | % At | E_B_ [eV] | % At | E_B_ [eV] | % At | E_B_  [eV] | % At | E_B_  [eV] | % At | E_B_  [eV] | % At | E_B_  [eV[ | % At |
| C 1s regions | | | | | | | | | | | | | | | | | |
| C 1s A | C=C sp^2^ | 284.20 | 24.1 | 284.21 | 24.8 | 284.13 | 23.9 | 284.12 | 28.4 | 284.15 | 10.1 | 284.16 | 9.5 | 284.39 | 12.2 | 284.45 | 9.7 |
| C 1s B | C-C sp^3^ | 284.77 | 41.0 | 284.78 | 40.5 | 284.69 | 43.9 | 284.66 | 41.9 | 284.74 | 58.2 | 284.71 | 61.7 | 284.80 | 57.9 | 284.74 | 70.2 |
| C 1s C | C-OH | 286.02 | 13.6 | 286.02 | 12.8 | 285.93 | 13.8 | 285.93 | 12.6 | 285.91 | 8.1 | 285.94 | 7.9 | 286.05 | 9.4 | 286.04 | 3.9 |
| C 1s D | C-O-C | 286.77 | 6.8 | 286.83 | 6.6 | 286.63 | 4.4 | 286.64 | 3.9 | 286.59 | 7.1 | 286.57 | 6.5 | 286.74 | 4.3 | 286.65 | 3.4 |
| C 1s E | C=O | 287.73 | 3.9 | 287.76 | 3.9 | 287.64 | 2.5 | 287.64 | 2.8 | 287.90 | 4.4 | 287.94 | 5.2 | 287.69 | 2.9 | 287.69 | 1.4 |
| C 1s F | O-C=O | 288.71 | 3.7 | 288.70 | 4.0 | 288.58 | 3.8 | 288.59 | 3.8 | 288.77 | 3.0 | 288.75 | 2.6 | 288.65 | 4.1 | 288.62 | 2.5 |
| C 1s G | CO_3_^2-^ | 289.32 | 0.6 | 289.30 | 0.9 | 289.22 | 0.6 | 289.22 | 0.5 | 289.26 | 0.4 | 289.34 | 0.3 | 289.70 | 1.1 | 289.23 | 0.8 |
| C 1s H | C-N | 285.44 | 6.3 | 285.42 | 6.6 | 285.35 | 7.1 | 285.35 | 6.2 | 285.35 | 8.8 | 285.35 | 6.3 | 285.41 | 8.1 | 285.40 | 8.0 |
| O 1s regions | | | | | | | | | | | | | | | | | |
| O 1s A | O-C=O | 530.92 | 10.0 | 530.97 | 8.3 | 530.91 | 7.3 | 530.98 | 9.8 | 529.97 | 3.2 | 529.99 | 3.4 | 530.80 | 3.9 | 530.97 | 5.2 |
| O 1s B | O=C | 531.73 | 30.9 | 531.71 | 28.2 | 531.65 | 28.3 | 531.71 | 33.1 | 531.19 | 20.9 | 531.15 | 22.6 | 531.63 | 29.9 | 531.71 | 30.1 |
| O 1s C | HO-C_alip_,  C-(O)-C_epoxy_ | 532.48 | 34.3 | 532.49 | 40.8 | 532.49 | 44.7 | 532.52 | 43.4 | 532.03 | 52.9 | 532.02 | 57.1 | 532.36 | 45.4 | 532.32 | 46.7 |
| O 1s D | HO-C_arom_, C-O-C | 533.37 | 20.2 | 533.52 | 17.9 | 533.30 | 18.0 | 533.36 | 12.9 | 532.85 | 16.5 | 532.98 | 12.1 | 533.39 | 19.0 | 533.24 | 16.7 |
| O 1s E | O-C=O | 534.46 | 4.4 | 534.41 | 3.5 | 534.36 | 1.8 | 534.26 | 0.8 | 533.75 | 5.0 | 533.78 | 3.3 | 534.58 | 1.8 | 534.15 | 1.3 |
| O 1s F | H_2_O | 535.75 | 0.3 | 536.00 | 1.3 | - | - | - | - | 535.42 | 1.6 | 535.71 | 1.6 | - | - | - | - |

E_B_ - binding energy; % At - atomic percentage
